# Successful COVID-19 rescue therapy by extra-corporeal membrane oxygenation (ECMO) for respiratory failure: a case report

**DOI:** 10.1186/s13037-020-00245-7

**Published:** 2020-05-08

**Authors:** Michael S. Firstenberg, Philip F. Stahel, Jennifer Hanna, Chakradhar Kotaru, Joseph Crossno, Joseph Forrester

**Affiliations:** 1 The Medical Center of Aurora, 1501 South Potomac St, Aurora, CO 80012 USA; 2 Cardiothoracic and Vascular Surgery, The Medical Center of Aurora, 1444 S. Potomac Street, Suite 200, Aurora, CO 80012 USA; 3 Department of Specialty Medicine, College of Osteopathic Medicine, Rocky Vista University, Parker, CO 80134 USA

**Keywords:** Coronavirus, COVID-19, SARS-CoV-2, Extra-corporeal membrane oxygenation, Lung injury, Remdesivir treatment

## Abstract

**Background:**

The value of extracorporeal membrane oxygenation (ECMO) for patients suffering from novel coronavirus disease 2019 (COVID-19) as a rescue therapy for respiratory failure remains controversial and associated with high mortality rates of 50 to 82% in early reports from Wuhan, China. We hypothesized that patient outcomes would be improved at our tertiary cardiothoracic surgery referral center with a protocolized team-approach for ECMO treatment of patients with severe COVID-19 disease.

**Case presentation:**

A 51-year-old healthy female developed severe acute respiratory syndrome coronavirus 2 (SARS-CoV-2) bilateral pneumonia while vacationing in Colorado with her family. She was transferred to our facility for a higher level of care. Her respiratory status continued to deteriorate despite maximized critical care, including prone positioning ventilation and nitric oxide inhalation therapy. Veno-venous ECMO was initiated on hospital day 7 in conjunction with a 10-day course of compassionate use antiviral treatment with remdesivir. The patient’s condition improved significantly and she was decannulated from ECMO on hospital day 17 (ECMO day 11). She was successfully extubated and eventually discharged to rehabilitation on hospital day 28.

**Conclusion:**

This case report demonstrates a positive outcome in a young patient with COVID-19 treated by the judicious application of ECMO in conjunction with compassionate use antiviral treatment (remdesivir). Future prospective multi-center studies are needed to validate these findings in a larger cohort of patients.

## Background

The novel coronavirus disease 2019 (COVID-19) global pandemic is associated with a high estimated case-fatality rate of around 3.5% [1–5]. The main clinical feature of severe acute respiratory syndrome coronavirus 2 (SARS-CoV-2) infection is represented by bilateral pneumonia with radiological signs of ground glass opacification [6]. One third of symptomatic COVID-19 patients require intensive care unit (ICU) admission and around 30% of these patients show signs of acute respiratory distress syndrome (ARDS) [6]. The high mortality of SARS-CoV-2 pneumonia is associated with intractable respiratory failure in up to 98% of all non-survivors, based on retrospective observational cohort studies from Wuhan, China [7, 8]. Extra-corporeal membrane oxygenation (ECMO) represents a potentially life-saving modality for patients with terminal respiratory failure [9]. However, early experiences from COVID-19 “hot zones” in China reported a high mortality rate of 50–82% in adult patients with ARDS from SARS-CoV-2 pneumonia [10, 11]. In the present case report, we describe the successful rescue treatment of a critically ill patient with respiratory failure due to COVID-19 and discuss the critical variables for effective ECMO therapy in this highly vulnerable patient cohort.

## Case presentation

A 51-year-old female travelled from Illinois to Colorado for a winter vacation with her family. She was a previous smoker (< 1 pack/day) and had been abstinent for more than 10 years. Past medical history consisted of a remote Caesarean section, medically controlled hypertension, no know allergies, and minimal social alcohol consumption. On March 6, 2020, she presented with an unstable ankle fracture to a local hospital and underwent surgery by open reduction with internal fixation the following day. Prior to her surgery, she had reported “mild cold symptoms”. Upon awakening from general anesthesia, during extubation she was noted to have an unusual consistency of her respiratory secretions described as “pink and frothy”. Empirical broad-spectrum antibiotics were initiated for the treatment of a presumed aspiration pneumonitis. The patient deteriorated over the subsequent days with fever, hypoxemia, and bilateral pulmonary infiltrates. She required intubation and mechanical ventilation. COVID-19 testing was performed by nasopharyngeal swab and was positive for SARS-CoV-2 by polymerase chain reaction (PCR). The patient was transferred to our center, an established ECMO program, for higher level of care 1 week after her initial admission.

The timeline of the patient’s course of events is summarized in Table 1.

**Table 1:** Summary of the timeline of events

Date	Event	Hospital day #	ECMO day #
March 6, 2020	Ankle fracture		
March 7, 2020	ORIF ankle fracture		
March 13, 2020	Transfer to our facility	1	
March 15, 2020	Nitric oxide and prone therapy initiated	3	
March 19, 2020	ECMO initiated	7	1
March 21, 2020	Remdesivir initiated	9	3
March 29, 2020	ECMO decannulation	17	11
March 31, 2020	Liberated from invasive mechanical ventilation	19	13
April 2, 2020	Transfer to floor, transition to 2 l nasal cannula	21	15
April 9, 2020	Discharge to rehabilitation	28	22

Abbreviations: *ECMO* Veno-venous extracorporeal membrane oxygenation, *ORIF* Open reduction with internal fixation

**Fig. 1:**
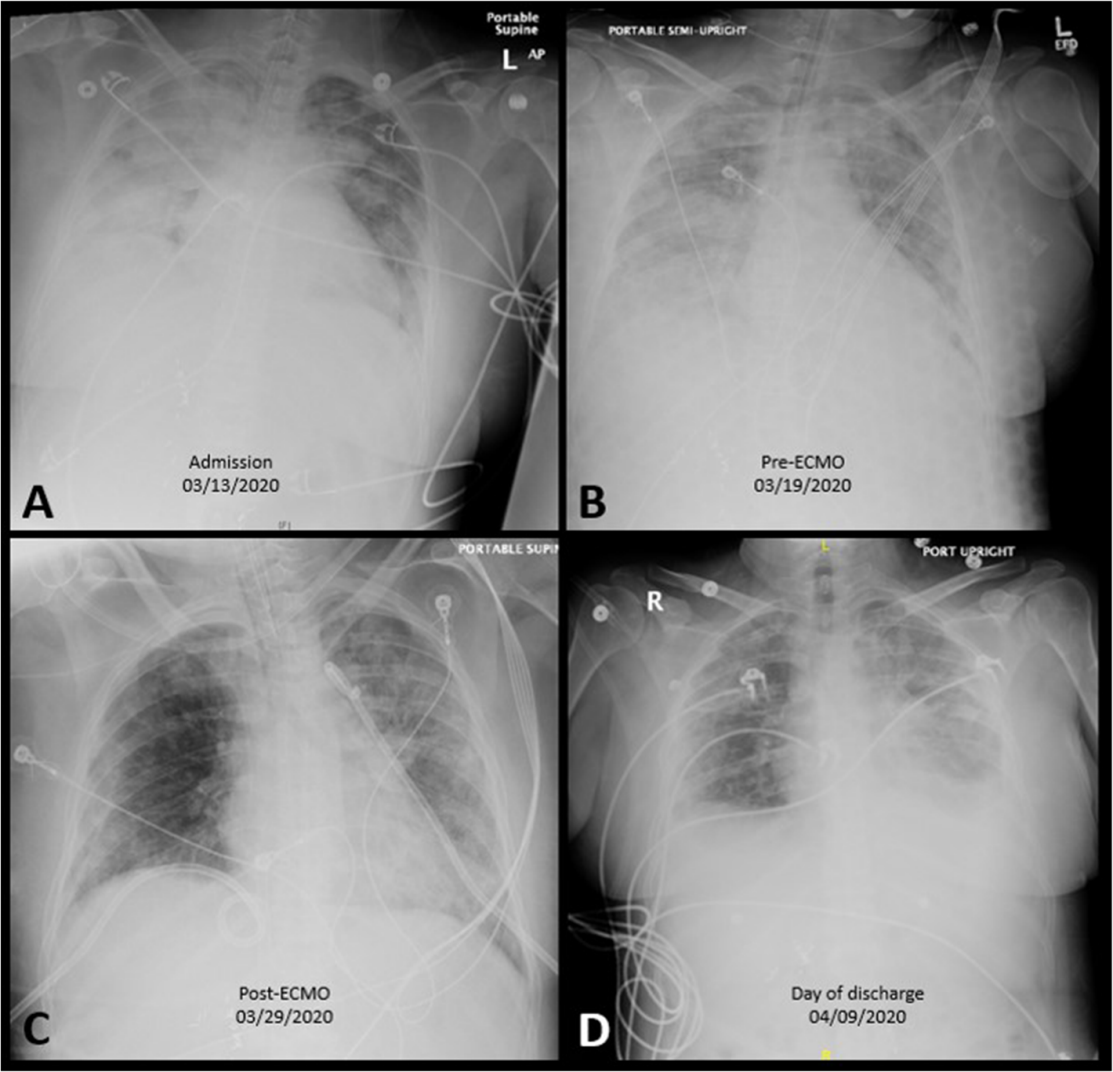
The patient’s chest X-rays on the day of admission (**a**); day of ECMO cannulation (**b**); day of ECMO decannulation (**c**); and day of discharge to rehabilitation (**d**)

Upon arrival to our facility, she was hemodynamically stable, sedated, intubated and mechanically ventilated on 60% FiO_2_ and 10 cm H_2_O positive end-expiratory pressure (PEEP). Figure 1 demonstrates the patient’s chest X-ray on admission. She was admitted to our designated COVID-cohorting unit in the ICU and placed with strict respiratory droplet isolation precautions in a negative airflow room. Her admission laboratory studies demonstrated a low white blood cell count of 3110/μL with lymphocytopenia (22.5% or 700/μL). A bedside transthoracic echocardiogram showed normal left and right ventricular function, no valvular abnormalities, and normal estimated pulmonary artery pressures. By hospital day 2, her oxygen requirements increased and intermittent prone positioning was initiated in conjunction with pharmacologic paralysis and inhaled nitric oxide treatment.

Within the next few days, the patient’s pulmonary compliance and hypoxemia worsened. Isolated end-organ pulmonary dysfunction persisted despite maximal ventilator support resulting in a PaO_2_/FiO_2_ ratio of 66 while still receiving inhaled nitric oxide iNO at 40 ppm. The arterial blood gas analysis (ABGA) showed a pH 7.36, pCO_2_ 45 mmHg, and pO_2_ 66 mmHg. The patient had a calculated “Respiratory ECMO Survival Prediction” (RESP) score of 1 with a risk class III and an estimated ECMO survival probability of 57% [12].

Percutaneous femoral-femoral veno-venous ECMO support was initiated on day 7 of hospitalization. In brief, following systemic heparinization (10,000 units; maintenance target PTT 40–60 s), cannulation was accomplished with a 21-French venous multi-stage drain and a 21-French single-stage inflow cannula connected to a CardioHelp™ extra-corporeal support system (Maquet Cardiovascular, Wayne, NJ). Initial ECMO settings were 3.8 l/min flow, 3.2 l of gas sweep, and ECMO FiO_2_ of 1.0. A lung protective ventilator strategy was initiated with an airway pressure release ventilation (APRV) mode (rate: 16, pressures: 28/10, FiO_2_: 0.8, inhaled nitric: 20 ppm, and plateau pressures were limited to 30 mmHg). Antiviral therapy with remdesivir (RDV / GS-5734; Gilead Sciences Inc., Foster City, CA) was initiated on hospital day 9 under a humanitarian use protocol for “compassionate use” indication [13], with a loading dose of 200 mg RDV intravenous on the first day, and once-daily maintenance doses of 100 mg RDV intravenous for a total treatment duration of 10 days.

A repeat echocardiogram showed no change in her left or right ventricular size or function. A bedside ultrasound demonstrated increasing bilateral pleural effusions which were managed by percutaneous drainage with bilateral 14-French chest tubes. The patient’s pulmonary compliance, oxygenation, and ventilation, gradually improved over the subsequent days, and the ECMO sweep was slowly weaned (ventilator settings at pressure control; mean airway: 25 mmHg; rate 25; FiO_2_ 0.50; inhaled nitric oxide 20 ppm). Subsequently, the ECMO gas sweep was weaned off and the patient was successfully decannulated at the bedside on ECMO day 11 (hospital day 17). Her overall pulmonary function improved to the point in which she was extubated to heated high-flow oxygen. The patient’s oxygen requirements were weaned to 2 l nasal cannula on post-ECMO day 4 (hospital day 21). Room air blood gas analysis revealed pH 7.46; pCO_2_ 33.6 mmHg; pO_2_ 74.4 mmHg; HCO_3_–23 mEq/L, SO_2_ 94%. A limited bedside spirometry yielded a negative inspiratory force (NIF) of − 50 mmHg.

**Fig. 2:**
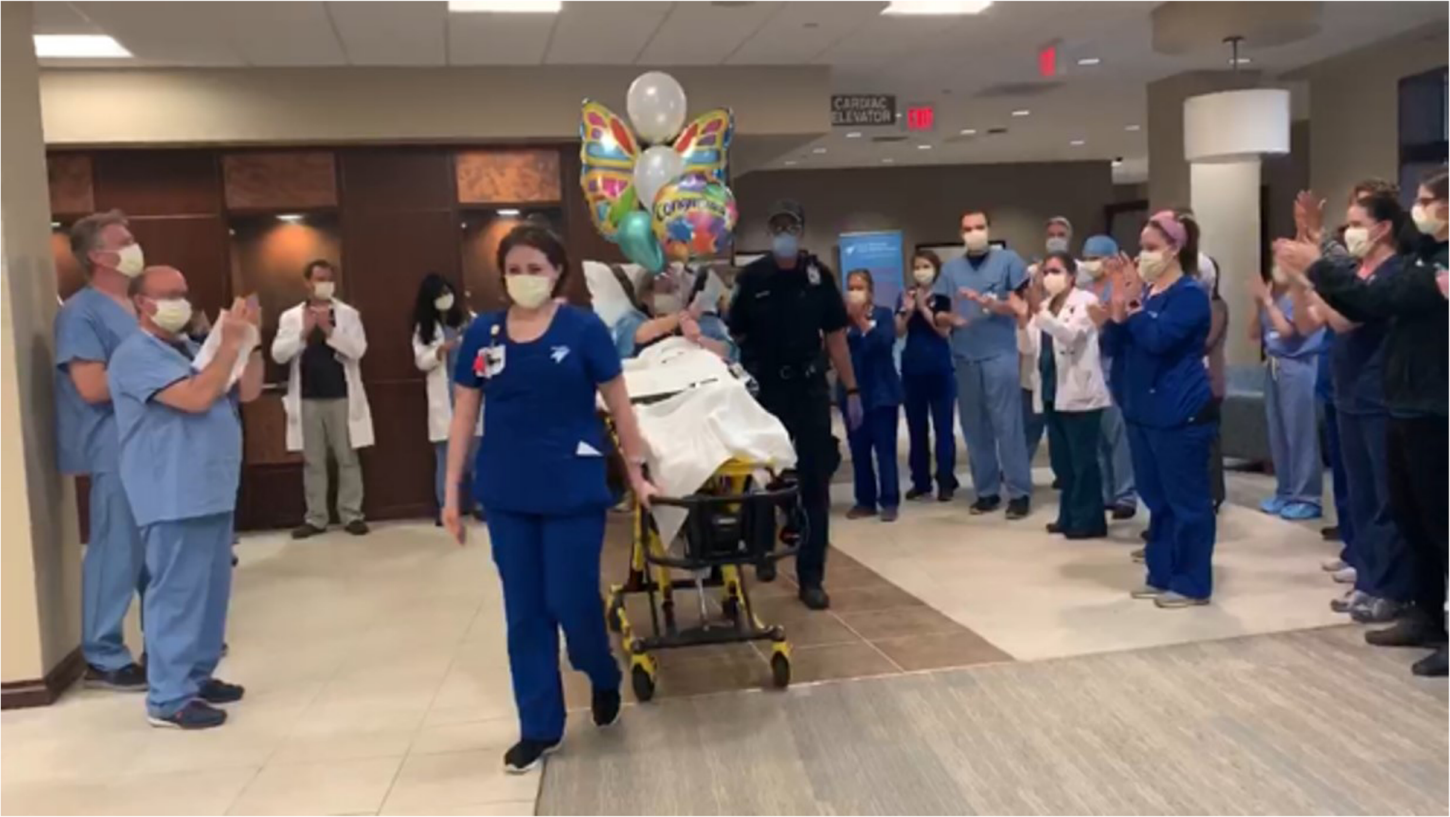
Farewell reception by hospital staff at the Medical Center of Aurora to the COVID-19 survivor discharging to rehabilitation on hospital day 28

Two repeat COVID-19 tests were negative for SARS-CoV-2 prior to discharge form the hospital. The patient was discharged to a rehabilitation facility on hospital day 28 requiring 2–3 l/minute of supplement oxygen which was weaned to room air within the week. At discharge, she was weak but able to stand and walk short distances and had an otherwise normal neurologic examination. Upon discharge she was greeted by a large group of health care providers as part of a farewell ceremony for our COVID-19 survivor (Fig. 2).

## Discussion

The role of ECMO for rescue therapy of respiratory failure in critically ill COVID-19 patients remains controversial [10, 11, 14, 15]. In this case report, we present a successful ECMO salvage case in a 51-year-old patient with bilateral SARS-CoV-2 pneumonia and refractory respiratory failure. We illustrate the technical aspects and crucial variables related to the indication and the ultimate positive outcome of this patient. Of note, our ECMO program consists of a multi-disciplinary team of specialists in which all major clinical decisions on ECMO patients are discussed during comprehensive twice daily team rounds attended by intensivists, cardiac surgeons, perfusionists, critical care nurses, pharmacy, and respiratory therapy, as previously described [16]. Monitoring of the ECMO circuit is performed by the perfusion team with 24/7 in-house coverage [16]. The current literature established a consensus that optimal outcomes in patients with profound respiratory failure and ARDS are achieved at specialized centers with full institutional commitment to advanced respiratory therapies [17].

**Fig. 3:**
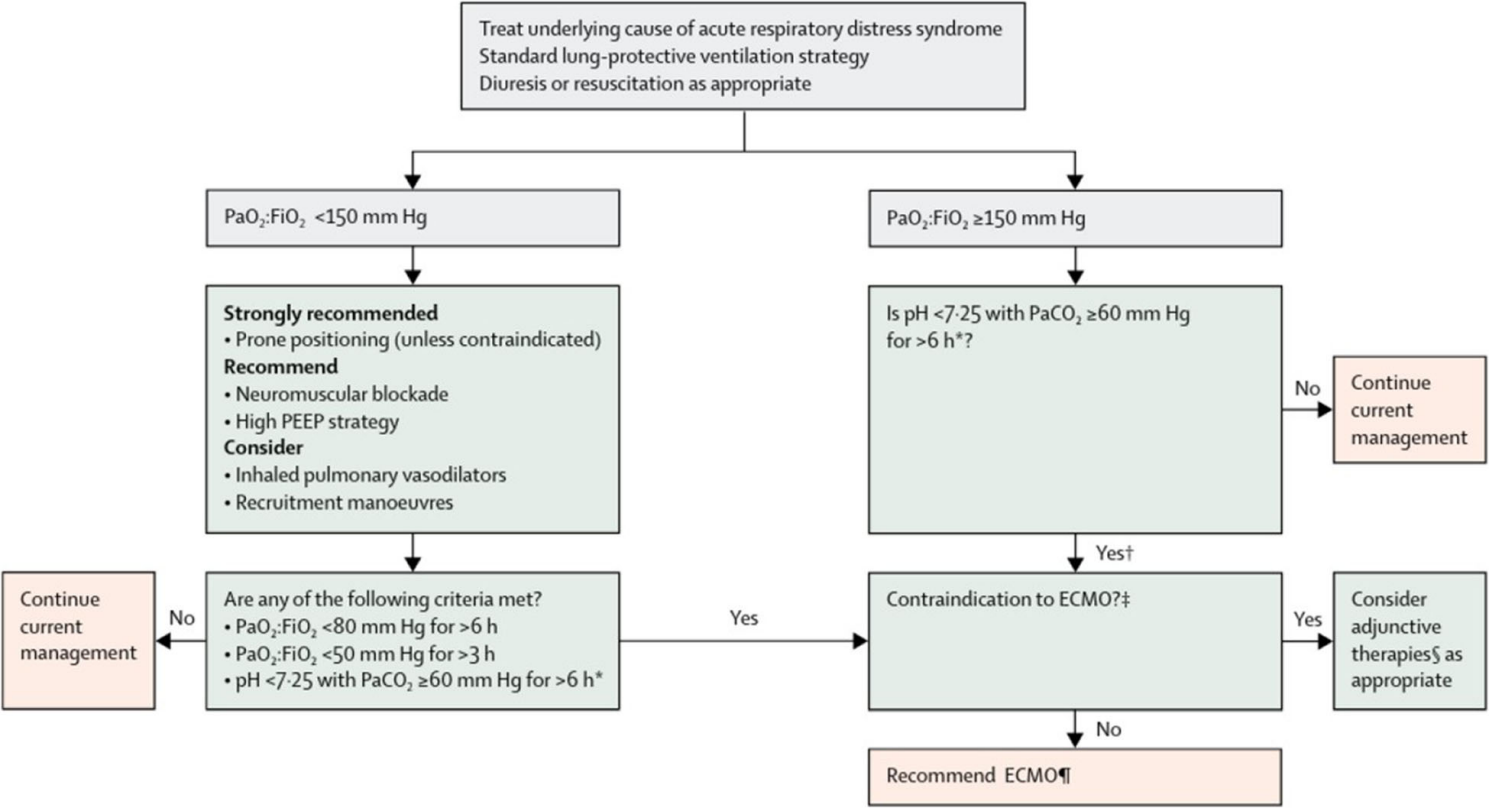
Algorithm for consideration of ECMO in patients with acute respiratory failure. Legend and abbreviations: PEEP = positive end-expiratory pressure. PaO_2_:FiO_2_ = ratio of partial pressure of oxygen in arterial blood to the fractional concentration of oxygen in inspired air. EMCO = extracorporeal membrane oxygenation. PaCO_2_ = partial pressure of carbon dioxide in arterial blood. *With respiratory rate increased to 35 breaths per minute and mechanical ventilation settings adjusted to keep a plateau airway pressure of < 32 cm of water. ^†^Consider neuromuscular blockade. ^‡^There are no absolute contraindications that are agreed upon except end-stage respiratory failure when lung transplantation will not be considered; exclusion criteria used in the EOLIA trial^1^ can be taken as a conservative approach to contraindications to EMCO. §Eg, neuromuscular blockade, high PEEP strategy, inhaled pulmonary vasodilators, recruitment manoeuvres, high-frequency oscillatory ventilation. ¶Recommend early ECMO as per EOLIA trial criteria; salvage ECMO, which involves deferral of ECMO initiation until further decompensation (as in the crossovers to ECMO in the EOLIA control group), is not supported by the evidence but might be preferable to not initiating ECMO at all in such patients. This algorithm is endorsed by the Extracorporeal Life Support Organization (ELSO) [18]. Reprinted with permissions from [17]: Abrams D, Ferguson ND, Brochard L, Fan E, Mercat A, Combes A, Pellegrino V, Schmidt M, Slutsky AS, Brodie D. ECMO for ARDS: from salvage to standard of care? *Lancet Respir. Med.* 2019, 7:108–10. (Reprint permission to Philip F. Stahel, MD: license # 4804540769693, April 8, 2020)

Consistent with current guidelines by the Extracorporeal Life Support Organization (ELSO), our institution has embraced the concept that ECMO should only be considered at programs that have established and dedicated programs [18]. Figure 3 demonstrates the decision-making algorithm for ECMO consideration, endorsed by the ELSO guidelines [17, 18]. In addition to the ELSO guidelines we utilize the RESP score developed by ELSO to determine optimal candidates for ECMO intervention. Conversely, while salvage therapies might be considered outside of an existing program, in part due to the poor outcomes associated with this approach, centers are discouraged from embarking on ECMO program development at this time for the singular purpose of providing support for COVID-19 patients [18, 19]. Another key component of the successful outcome in the patient presented in this report is the timing of the initiation of therapy. While the literature reports varying thresholds in which veno-venous ECMO should be considered for acute respiratory failure, the general indications are profound respiratory failure refractory to conventional medical and ventilator management [9, 20, 21]. This includes preceding attempts for “salvage” therapies with inhaled nitric oxide, prone positioning ventilation, and judicious use of paralytic agents [9, 20, 21]. Patients with profound hypoxemia, hypercapnia, respiratory acidosis, and worsening pulmonary compliance in the absence of mechanical causes are typically considered ECMO candidates [9, 20, 21]. Contraindications to ECMO include concerns for acute neurologic insults, such as anoxic brain injury, contraindications to anticoagulation, and baseline comorbidities that predict either futility of ECMO or limited 2-year survival [9, 20, 21]. Current indications for ECMO in COVID-19 patients were recently proposed based on the early experience with the pandemic in China [22, 23]. As the pandemic spreads and resources (such as ECMO circuits) become limited, we advocate potentially limiting ECMO to those patients who have the best predicted outcomes. While the Survival After Veno-arterial (SAVE) and RESP scores [12] are helpful in selecting patients, they do not inherently account for poor ECMO outcomes such as acute or worsening end-organ damage and advanced age. We advocate ECMO for those patients with isolated single organ (pulmonary) dysfunction, few co-morbidities, and limited acute – and potentially reversible – end-organ dysfunction. Appropriate candidates should also probably be relatively young and otherwise hemodynamically stable.

As shown in this case report, the most crucial aspect to initiating therapy is the timing between reaching the threshold for indications and the decision to start therapy. While it is reasonable to consider adjuvant therapies – like inhaled nitric oxide and prone positioning ventilation, the care team must define clear thresholds for when the potential success of these adjunctive therapies may be considered exhausted and ECMO therapy should be considered. In the context of COVID-19 patients whose respiratory capacity can deteriorate very quickly, the decision to initiate therapy must be anticipated in advance and before the onset of potentially irreversible end-organ dysfunction. Unequivocal thresholds for considering ECMO are particularly important in this current COVID-19 pandemic as concerns of limited resources are prompting discussions of rationing therapies to those who might benefit the most from their use [24]. It is also important to realize that ECMO might have other potential benefits beyond supporting oxygenation and ventilation needs. When configured in a veno-arterial mode, cardiac output for patients in acute systolic heart failure can be supported [20, 21]. Since COVID-19 is also associated with hyperpyrexia and cytokine storm, all of which can increased metabolic and oxygenation requirements, ECMO may also play a role in active quiescence of COVID-19 patients [18]. Preliminary United States Food and Drug Administration (FDA) authorization has been given to special “ECMO filters” that might assist in viral clearance (www.fda.gov/media/136867/download).

Various other root causes can potentially contribute to the poor outcomes associated with ECMO use in SARS-CoV-2 infected patients [23]. Health care facilities that are not experienced in the management of these extremely ill and complex patients, especially if their respective experience is restricted to a few select patients, may have worse outcomes than established tertiary-referral ECMO centers [16]. This idea of referral to specialized ECMO centers also feeds into the concerns of patient selection and the timing of initiation of therapy – delay in initiation of ECMO might predispose to a potentially worse outcome as the patient might have already reached a “point of no return” with regard to irreversible pathophysiology and evolving multi-organ dysfunction.

Concerns were recently raised that ECMO may contribute to the lymphopenia observed in COVID-19 patients thereby deteriorating the patient’s cellular immune response and ability to clear the SRAS-CoV-2 pathogen effectively [14]. The extra-corporeal circuit has been shown to exaggerate an already dysfunctional immune system [25] and this adverse effect may negatively impact the outcomes of COVID-19 patients [14]. The role of immune-modulating therapies in the setting of advanced COVID-19 infections in mitigating the “cytokine storm” leading to hyperinflammation and adverse outcomes is a topic of intense ongoing investigation [25–28].

It is imperative to understand that ECMO neither treats nor cures a disease, as the fundamental principle of ECMO consists of allowing the lungs to “rest” while the primary therapy, such as antiviral treatment, can take effect with less concern for ventilator-induced lung injury [29, 30]. This notion is confirmed in the current case report, where the beneficial adjunctive effects of ECMO likely supported the patient’s viral clearance by the antiviral therapy through the compassionate use indication for remdesivir [13]. An additional benefit of ECMO is to provide improved tissue oxygenation, carbon dioxide clearance, and systemic acid-base balance resolution to avoid end-organ physiologic shock, all of which are significant causes of mortality in the COVID-19 patient [31].

Fundamentally, ECMO should never be considered a primary therapy for any form of acute lung injury, but rather utilized as a lung-protective adjuvant modality that promotes a physiological respite and a “biological milieu” for lung healing and recovery. While the scientific evidence for the benefit of ECMO therapy in patients with severe COVID-19 still awaits further validation [32], the consideration of early referral of patients with respiratory failure from SARS-CoV-2 pneumonia to designated centers of excellence appears justified from a patient safety perspective.

## Conclusion

This case report demonstrates a positive outcome in a young, critically ill patient after judicious application of ECMO for respiratory failure due to SARS-CoV-2 pneumonia. If ECMO is considered as a treatment for COVID-19, we suggest timely referral to a tertiary center with established expertise and standardized ECMO protocols. Future prospective multi-center studies are needed to validate these findings in a larger cohort of patients.

## Data Availability

Please contact the author for data requests.
